# Supplementary material for: Identification of photoperiod-induced specific miRNAs in the adrenal glands of Sunite sheep (Ovis aries)
Source: Front Vet Sci. 2022 Jul 22;9:888207. doi: 10.3389/fvets.2022.888207 (PMC9354845; doi:10.3389/fvets.2022.888207)
Supplement: Supplementary file 1 [file Data_Sheet_1.ZIP › Supplementary file/miRDeep2 file.zip!/miRDeep2 file/Novel_Structure/SP42A1/Novel_120.pdf]

Provisional ID	:	NC_019459.2_4150
Score total	:	1.2
Score for star read(s)	:	-1.3
Score for read counts	:	0
Score for mfe	:	0.9
Score for randfold	:	1.6
Score for cons. seed	:	
Total read count	:	16
Mature read count	:	16
Loop read count	:	0
Star read count	:	0

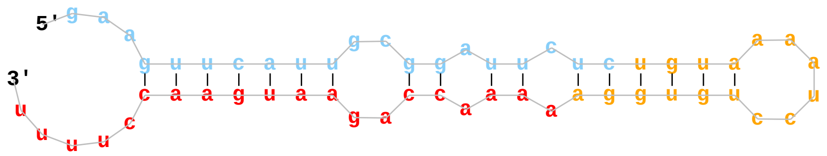

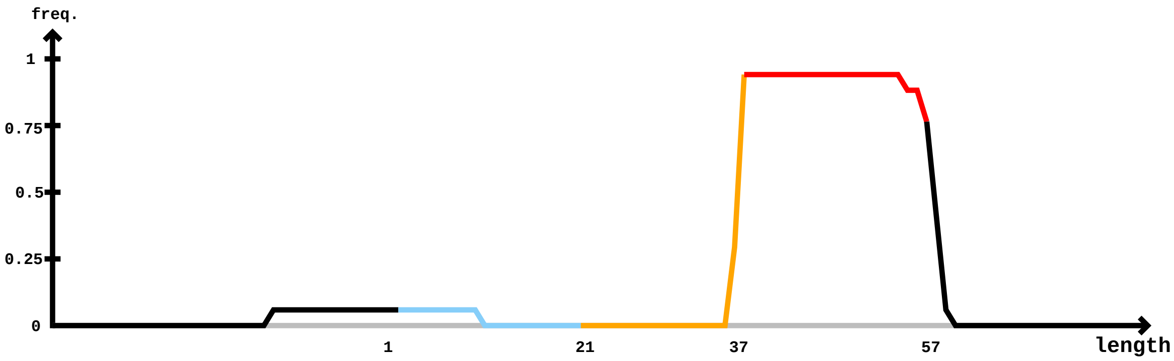

Star Mature

```
5'- aagaaaacaauacucauugcauuggguuggccaagaaguucauugcggauucucuguaaaauccuguggaaaaaccagaaugaaccuuuugaccaacccaguaccauuguug  -3'   exp
    ..............((.((.(((((((((.(((((.(((((((..((.((.((((((......)))))).)).))..))))))).))))).)))))))))..)).))...   reads    mm      sample
    .....................uuggguuggccaaCaaguucau.....................................................................   1        1       seq
    .....................................................................aaaaaaccUgaaugaaccu.........................   1        1       seq
    .....................................................................aaaaaaccagaaugaacUuuuu......................   2        1       seq
    .....................................................................aaaaaaccUgaaugaaccuuuu......................   2        1       seq
    ......................................................................aaaaccagaaugaacUuuu.......................   2        1       seq
    ......................................................................aaaaccagaaugaacUuuuu......................   4        1       seq
    ......................................................................aaaaccUgaaugaaccuuuu......................   1        1       seq
    ......................................................................aaaaccUgaaugaaccuuuug.....................   1        1       seq
    ......................................................................aaaaccagaaugaacUuuuug.....................   2        1       seq
    ......................................................................aaaaccagaaugaaccuuuuga....................   1        0       seq
```
